# Computer-assisted cognitive remediation therapy increases hippocampal volume in patients with schizophrenia: a randomized controlled trial

**DOI:** 10.1186/s12888-018-1667-1

**Published:** 2018-03-27

**Authors:** Tsubasa Morimoto, Yasuhiro Matsuda, Kiwamu Matsuoka, Fumihiko Yasuno, Emi Ikebuchi, Hiroyuki Kameda, Toshiaki Taoka, Toshiteru Miyasaka, Kimihiko Kichikawa, Toshifumi Kishimoto

**Affiliations:** 1 Department of Psychiatry, Nara Medical University School of Medicine, 840 Shijo-cho, Kashihara, Nara, 634-8522 Japan; 2 Department of Psychiatry, Teikyo University School of Medicine, 2-11-1 Kaga, Itabashi-ku, Tokyo, 173-8605 Japan; 3 School of Computer Science, Tokyo University of Technology, 1404-1 Katakuramachi, Hachioji City, Tokyo, 192-0982 Japan; 4 Department of Radiology, Nagoya University Hospital, 65 Tsurumai-cho, Showa-ku, Nagoya, Aichi 466-8550 Japan; 5 Department of Radiology, Nara Medical University School of Medicine, 840 Shijo-cho, Kashihara, Nara, 634-8522 Japan

**Keywords:** Cognitive remediation, Rehabilitation, Structural neuroimaging, Hippocampus, Schizophrenia, Jcores

## Abstract

**Background:**

Cognitive remediation therapy (CRT) effectively reduces neurocognitive impairment in patients with schizophrenia, but few studies have used structural neuroimaging methods to assess its neuroanatomical effects. We investigated these effects, as well as the association between changes in cortical volume and neurocognitive performance.

**Method:**

Between August 2013 and September 2016, we performed a randomized controlled study comprising a CRT group (16 individuals) and a treatment-as-usual (TAU) group (15 individuals) of patients with schizophrenia. CRT participants engaged in twice-weekly computer-assisted CRT sessions and weekly group meetings for 12 weeks. T1-weighted magnetic resonance imaging was performed before and after the intervention period, and whole-brain voxel-based morphometric analysis was used to detect significant cortical gray matter volume changes. We also assessed the correlation between cortical volume changes and CRT-derived neurocognitive improvements.

**Results:**

The CRT group exhibited significantly greater improvements than the TAU group in verbal fluency (*P* = 0.012) and global cognitive scores (*P* = 0.049). The CRT group also exhibited significantly greater increases in right hippocampal volume than the TAU group (*P* < 0.001). Changes in verbal fluency scores and right hippocampal volumes were positively correlated (*r* = 0.53, *P* = 0.001).

**Conclusion:**

We found that CRT significantly increased right hippocampal volumes and that these enhancements were positively correlated with changes in verbal fluency scores. Our results indicate that CRT induces cognitive improvement through hippocampal plasticity.

**Trial registration:**

Registration number: UMIN000026146, 2017/02/15, retrospectively registered.

## Background

Kraeplin referred to schizophrenia as “dementia praecox” and described it as a progressive neurodegenerative disease resulting in cognitive deficits [1]. Indeed, studies addressing progressive neuropsychological changes in schizophrenia indicate that patients experience gradual decline in neurocognitive function [2]. Several structural magnetic resonance imaging (MRI) studies have reported progressive gray matter (GM) loss in the brain of individuals with schizophrenia. These findings were supported by a recent meta-analysis of 19 trials including 813 patients, which discovered significantly accelerated whole-brain GM volume loss in individuals with schizophrenia [3].

Cognitive impairment is an important aspect of schizophrenia. Therefore, possible means of alleviating cognitive deficits are of interest to clinicians and researchers. Because antipsychotics do not improve neurocognitive function as much as previously hoped [4, 5], researchers are considering alternative approaches such as cognitive remediation therapy (CRT) [6, 7], an evidence-based non-pharmacological treatment intended to improve functions related to daily tasks, including those related to school, work, social interactions, and independent living [8]. A meta-analysis of 26 randomized, controlled trials of CRT for schizophrenia treatment (totaling 1151 patients) reported that CRT has a medium effect size (0.41) on cognitive performance [9].

Over the last 15 years, investigators have been developing and optimizing computer-assisted CRT for individuals with schizophrenia [10]. We developed a program called the Japanese Cognitive Rehabilitation Program for Schizophrenia (Jcores). Recent studies have shown that a 12-week CRT program using Jcores (Vocational Cognitive Ability Training by Jcores [VCAT-J]) improves neurocognitive performance [11].

Nonetheless, it remains unknown how CRT mediates neuroanatomical changes. To date, two randomized, controlled studies have used structural neuroimaging to test the effect of CRT on individuals with schizophrenia. Penadés et al. [12] reported that, compared to social skills training, paper-and-pencil task CRT produces significantly greater improvements in the fractional anisotropy index of white matter in the corpus callosum and the right posterior thalamic radiation. Eack et al. [13] showed that computer-assisted CRT protects against GM loss in the hippocampus, parahippocampal gyrus, and left fusiform gyrus, as evidenced by a 2-year follow-up study that compared patients with and without CRT.

While the interventions developed by Penadés et al. [12] and Eack et al. [13] require over 4 months and 2 years, respectively, VCAT-J requires only 12 weeks. In the current study, we examined whether the VCAT-J could affect the brain structures of individuals with schizophrenia. We hypothesized that the VCAT-J intervention would induce changes in specific brain regions, especially the hippocampus—the volume of which is associated with various types of cognitive performance [14] and wherein activity-dependent neuroplasticity has repeatedly been observed [13, 15–18]. We also searched for associations between post-intervention regional cortical volume and neurocognitive functional changes, as assessed through scores on the Japanese version of the Brief Assessment of Cognition in Schizophrenia (BACS-J).

## Methods

### Participants and procedure

We recruited patients from one university hospital and two private hospitals by posters placed in the outpatient department and the day-treatment unit. Overseeing medical professionals have declared that all participants were capable of providing consent for themselves. To meet the selection criteria of the study, participants were required to: be of outpatient status, meet the criteria for schizophrenia established by the Diagnostic and Statistical Manual of Mental Disorders-Fourth Edition (Text Revision), and be aged between 20- and 60-years-old at the time of registration. Patients were excluded if they exhibited evidence of an organic central nervous system disorder, a history of drug or alcohol abuse, or intellectual disability.

Thirty-three patients consented to participate in this study, but one was excluded because of an organic disease (subdural hygroma), and another declined to undergo MRI because of possible pregnancy. A controlled, randomized study was conducted with two groups. Using a central registration system, participants were randomly assigned to either a CRT group (*n* = 16) that was treated immediately or a waitlist control group (*n* = 15) that continued treatment as usual (TAU) for 12 weeks before beginning CRT. All participants were followed up for 12 weeks, underwent T1-weighted MRI, and underwent clinical and neuropsychological assessments before and after intervention, which was performed by personnel blinded to the group assignments.

### Treatments

All participants were prescribed antipsychotics and received standard outpatient treatment. Participants in the CRT group completed computerized Jcores training, which exercises a broad range of cognitive functions, including attention, psychomotor speed, learning, verbal fluency, memory, and executive function. Each domain of the Jcores includes three to seven game types, and each game has five difficulty levels. Participants were asked to select specific cognitive domains and to adjust the task difficulty according to their abilities. For 12 weeks, the CRT group completed twice-weekly, hour-long computerized training sessions and weekly, hour-long group sessions, the purposes of which were to help establish associations between the computerized CRT and daily life or work performance as well as to aid in setting and tracking each participant’s individualized community life and/or work goals [7]. This study’s VCAT-J training procedures were identical to those of our previous study [11]. Psychiatrists or psychologists with computer-assisted CRT [6] study experience monitored all VCAT-J sessions.

TAU group participants primarily received medication and medical care from psychiatrists. Participants in the TAU group were also referred for social welfare and received assistance from a medical social worker as needed.

### Clinical assessments

Symptom severity was assessed using the Positive and Negative Syndrome Scale [19]. Neurocognitive function was assessed using the BACS-J, which has been validated as a reliable and practical evaluation of neurocognitive function in patients with schizophrenia [20]. The BACS-J tests verbal memory, working memory, motor speed, verbal fluency, attention and information processing speed, and executive function. Composite BACS-J scores were calculated by averaging z-scores from the six subcomponents, which were in turn calculated from the means and standard deviations extracted from a dataset of 64 healthy control Japanese individuals [21], age-matched to this study’s participants.

Functions related to daily life and community interactions were assessed using the Life Assessment Scale for the Mentally Ill [22], which measures a patient’s social skills in five categories of daily life: 1) daily living, 2) interpersonal relations, 3) work skills, 4) endurance and stability, and 5) self-recognition. Each category has several items that are rated on a five-point scale (0 = “no problem,” 4 = “serious problem”). In this study, we used scores from the interpersonal relations and work skills categories.

All neuropsychological assessments were evaluated by psychiatrists or psychologists uninvolved in CRT or regular outpatient treatment.

### MRI data acquisition

All MRI examinations were performed using a 3.0-T clinical scanner (Magnetom Verio; Siemens, Erlangen, Germany) with a 32-channel phased-array brain coil. High-resolution, three-dimensional T1-weighted images were acquired using a magnetization-prepared rapid gradient-echo sequence (repetition time = 1800 ms, echo time = 2.4 ms, flip angle = 10°, field of view = 256 mm; 208 sagittal plane sections; acquisition matrix = 256 × 256; acquired resolution = 1 × 1 × 1 mm^3^).

### Image processing

Image preprocessing and statistical analyses were performed with the SPM12 software (Wellcome Trust Centre for Neuroimaging, London, UK; http://www.fil.ion.ucl.ac.uk/spm), and voxel-based morphometry was performed using the Computational Anatomy Toolbox for SPM (CAT12; Jena University Hospital Departments of Psychiatry and Neurology, Jena, Germany; http://dbm.neuro.uni-jena.de/cat/) running on MATLAB R2014a (MathWorks, Natick, MA). We used the default preprocessing approach to analyze longitudinal data in CAT12. T1-weighted images were subjected to intra-subject realignment, bias correction, tissue segmentation into GM, white matter, and cerebrospinal fluid; and registration using linear (affine registration) and nonlinear transformations through Diffeomorphic Anatomical Registration Through Exponentiated Lie Algebra [23] within a unified model [24]. GM segments were subsequently analyzed by multiplying them by the nonlinear components derived from the normalization matrix to preserve actual local GM values (modulated GM volumes). Finally, the modulated and normalized GM segments (voxel size = 1.5 × 1.5 × 1.5 mm^3^) were smoothed using a Gaussian kernel of 8-mm full-width at half-maximum.

### Statistical analysis

We performed between-group comparisons of baseline demographic and clinical characteristics using two-sample student’s *t* and chi-square tests. Post-intervention changes in psychiatric symptoms, neurocognitive function, and social function were examined using analyses of variance (ANOVAs). We defined statistical significance as *P* < 0.05.

We performed an voxel-based analysis to investigate the relationship between post-CRT rehabilitation and GM volume changes, organizing a two-time (baseline versus 12-week follow-up) × two-group (CRT group versus TAU group) flexible factorial design in SPM12. We thus had four conditions: the CRT group at baseline, CRT group at follow-up, TAU group at baseline, and TAU group at follow-up. To test our hypothesis that rehabilitation would increase regional brain volumes, we investigated the interaction effect of increased post-rehabilitation volume by comparing volume increases in the CRT group to those in the TAU group. We used global scaling by total intracranial volume (TIV) to correct for different brain sizes. To avoid possible edge effects between different tissue types, we excluded all voxels outside the GM with absolute threshold masking. The mean TIV was 1619 cm^3^, and all images were globally scaled to a value of 50, creating an overall scaling of 50/1619. To obtain an absolute threshold of 0.2, we multiplied 0.2 by 50/1619. As this was a hypothesis-led analysis, we applied a liberal significance threshold of *P* < 0.001 with a 35-voxel whole-brain extent. Volumes of interest (VOIs) were determined from clusters where significant interactions were found. Regional volumes were calculated by averaging the values for all voxels within the VOIs. VOI volume changes over the intervention were compared between groups using repeated-measures ANOVAs.

We used Spearman’s correlation analysis to examine how VOI volume changes were related to various metrics in which the CRT group exhibited significantly greater improvements than the TAU group. Statistical analyses were performed using SPSS for Windows v. 21.0 (IBM, Armonk, NY).

## Results and statistical analyses

### Demographic and clinical data

Table 1 summarizes subject demographic and clinical characteristics. The CRT and TAU groups did not significantly differ in terms of demographics or baseline psychiatric symptoms, social function, or neurocognitive function.

### Changes in psychometric scores

As shown in Table 2, the CRT group exhibited significantly greater improvements in verbal fluency (F = 7.12, *P* = 0.012) and BACS-J composite scores (F = 4.21, *P* = 0.049) than the TAU group. No evidence of significant differences between groups were found regarding psychiatric symptoms or social function.

**Table 1:** Demographic data and baseline clinical characteristics

	CRT group (*n* = 16)		TAU group (*n* = 15)			
	Mean	SD	Mean	SD	*t* or χ^2^	*P*
Sex (male/female)	10/6		9/6		χ^2^ = 0.02	0.59
Age (years)	36.1	7.7	37.4	11.0	*t* = − 0.39	0.70
Handedness (right/left)	16/0		13/2		χ^2^ = 2.28	0.23
Years of education	14.3	2.4	13.0	2.0	*t* = 1.69	0.10
Years from onset of schizophrenia	12.1	7.8	13.9	13.2	*t* = −0.47	0.65
Number of hospitalizations	1.0	1.2	2.1	1.9	*t* = − 2.00	0.056
JART	102.6	11.8	97.8	12.2	*t* = 1.10	0.28
Drugs						
Mean dosage of antipsychotics^a^	440.3	265.1	680.3	530.9	*t* = −1.61	0.12
Mean dosage of anticholinergics^b^	0.75	1.65	1.30	1.51	*t* = −0.97	0.34
PANSS						
Positive symptoms	14.8	4.9	14.3	4.2	*t* = 0.26	0.80
Negative symptoms	20.7	4.4	20.6	7.2	*t* = 0.04	0.97
General psychopathology	39.6	7.8	41.5	10.5	*t* = −0.57	0.57
LASMI						
Interpersonal relations	11.1	3.9	11.5	7.9	*t* = −2.13	0.83
Work skills	10.6	3.4	12.6	5.7	*t* = −1.22	0.23
BACS (z-score)						
Verbal memory	−1.8	1.0	−2.0	1.1	*t* = 0.56	0.58
Working memory	−1.3	1.2	−1.3	1.2	*t* = −0.18	0.86
Motor speed	−2.4	1.4	−2.8	1.6	*t* = 0.76	0.45
Verbal fluency	−1.4	0.8	−0.9	0.8	*t* = − 1.68	0.10
Attention and speed of information processing	−1.6	0.8	−2.1	0.9	*t* = 1.45	0.16
Executive functions	−0.4	2.3	−1.1	1.6	*t* = 0.97	0.34
Composite score	−1.5	0.9	−1.7	0.9	*t* = 0.63	0.53

*Abbreviations*: *BACS* Brief Assessment of Cognition in Schizophrenia, *CRT* cognitive remediation therapy, *JART* National Adult Reading Test, Japanese Version, *LASMI* Life Assessment Scale for the Mentally Ill, *PANSS* Positive and Negative Syndrome Scale, *SD* standard deviation, *TAU* treatment as usual

^a^Chlorpromazine-equivalent dose (mg/day)

^b^Biperiden-equivalent dose (mg/day)

**Table 2:** ANOVAs of post-intervention clinical variable changes

	CRT group (*n* = 16)		TAU group (*n* = 15)			
	Mean	SD	Mean	SD	F	P
PANSS						
Positive symptoms	−0.88	2.16	0.33	3.09	1.61	0.21
Negative symptoms	−2.63	2.03	−0.67	5.04	2.06	0.16
General psychopathology	−2.38	4.13	0.40	8.17	1.45	0.24
LASMI						
Interpersonal relations	−1.75	2.18	−0.33	5.02	1.06	0.31
Work skills	−0.69	1.85	−0.33	1.29	0.38	0.54
BACS (z-score)						
Verbal memory	1.02	0.85	0.55	0.81	2.40	0.13
Working memory	0.24	0.66	−0.13	0.98	1.56	0.22
Motor speed	0.38	1.18	0.08	1.41	0.41	0.53
Verbal fluency	0.56	0.82	− 008	0.47	7.12	0.012
Attention and speed of information processing	0.25	0.52	0.04	0.50	1.33	0.26
Executive functions	0.48	1.85	0.42	1.37	0.01	0.92
Composite score	0.49	0.42	0.15	0.50	4.21	0.049

*Abbreviations*: *ANOVA* analysis of variance, *BACS* Brief Assessment of Cognition in Schizophrenia, *CRT* cognitive remediation therapy, *JART* National Adult Reading Test, Japanese Version, *LASMI* Life Assessment Scale for the Mentally Ill, *PANSS* Positive and Negative Syndrome Scale, *SD* standard deviation, *TAU* treatment as usual

### Increased regional GM volumes following rehabilitation

**Fig. 1:**
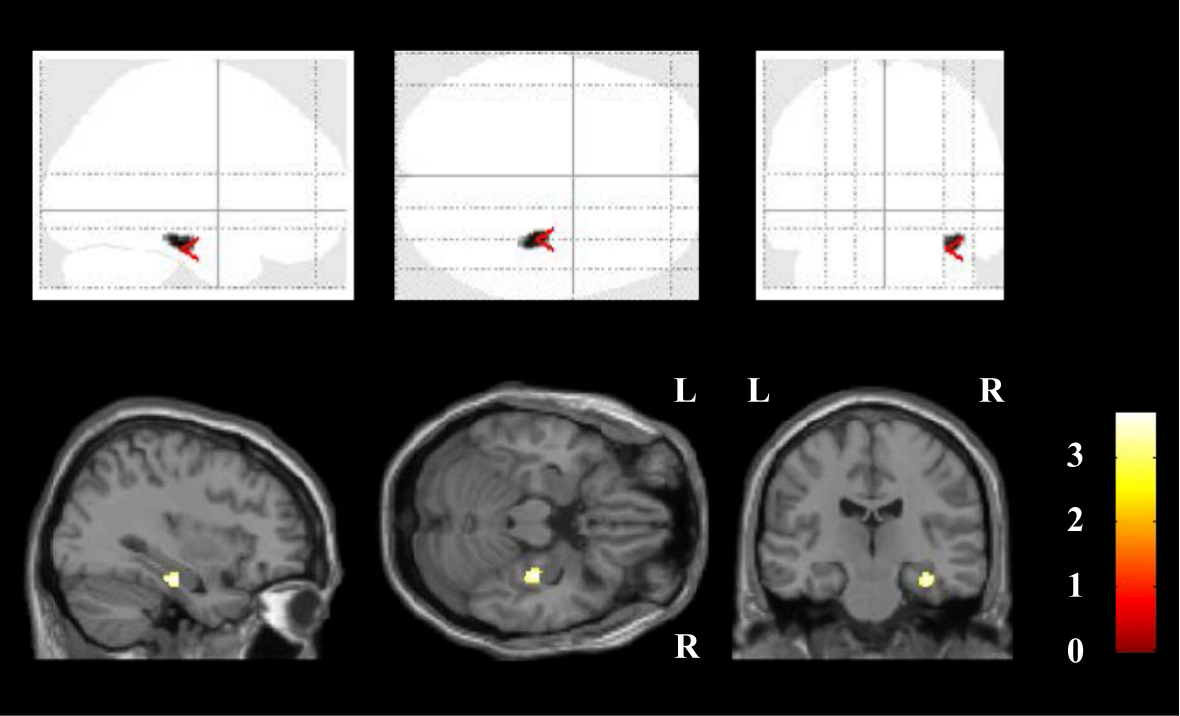
Regional GM increases among participants receiving CRT versus TAU. Increases in GM volume in the CRT and TAU groups, as assessed using voxel-based analysis. We detected areas where the CRT group’s GM volume growth was significantly greater than that of the TAU group (uncorrected *P* < .005 in 200 or more contiguous voxels). Statistical parametric mapping projections were then superimposed onto representative transaxial (z = − 21), sagittal (x = 35), and coronal (y = − 21) magnetic resonance images. Abbreviations: CRT, cognitive remediation therapy; GM, gray matter; L, left; R, right; TAU, treatment as usual

**Fig. 2:**
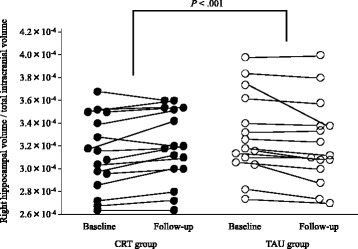
Changes in right hippocampal volume as a proportion of total intracranial volume. Scatterplots showing right hippocampal volume changes in the CRT and TAU groups. VOI clusters were placed where a voxel-based analysis indicated that the CRT group exhibited significantly greater volume increases than the TAU group. Abbreviations: CRT, cognitive remediation therapy; TAU, treatment as usual; VOI, volume of interest

Voxel-based analysis revealed that the CRT group exhibited significantly greater increases in right hippocampal GM volume than the TAU group ([x, y, z] = [35, − 21, − 21], cluster voxel size = 35, *T* = 3.70; Fig. 1). A repeated measures ANOVA revealed a significant group effect on right hippocampal and total intracranial raw volume changes (group-by-volume interaction, F (_1, 29_) = 16.1; *P* < 0.001, Fig. 2).

### Relationship between volume and psychometric scores

**Fig. 3:**
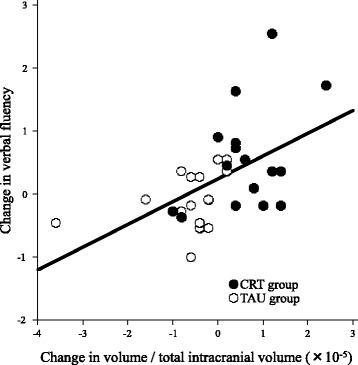
Association between changes in verbal fluency and right hippocampal volume in both groups. We observed a significant, positive correlation in the relationship between changes in verbal fluency and in raw right hippocampal volume as a proportion of total intracranial volume in the CRT and TAU groups (*r* = 0.53, *P* = 0.001, y = 0.25 + [3.6 × 10^4^] x). CRT, cognitive remediation therapy; TAU, treatment as usual

Next, we tested the relationship between hippocampal volume changes and changes in verbal fluency and BACS-J composite scores. We found that right hippocampal and total intracranial raw volume changes were positively correlated with verbal fluency improvements in both groups (*r* = 0.53, *P* = 0.001; Fig. 3), but no significant correlation was found with BACS-J composite score changes in either group.

## Discussion

This is the first study to show hippocampal volume increases in patients with schizophrenia following targeted CRT intervention. We found that 12 weeks of computer-assisted CRT produced significantly greater increases in right hippocampal GM volume than were accomplished with conventional TAU. One potential mechanism underlying this increase could be enhanced levels of brain-derived neurotrophic factor (BDNF), as Vinogradov et al. [25] reported that individuals with schizophrenia who engaged in a 10-week computerized cognitive training program showed significantly greater increases in serum BDNF than were observed in control subjects. BDNF plays a critical role in maintaining adult hippocampal volumes [26] by increasing axonal branching, dendrite complexity [27], spine density [28], and the number of neurons with hippocampal projections [26]. However, as we did not measure BDNF levels in the current study, we cannot confirm whether the observed increase in hippocampal GM volume is attributable to enhanced BDNF. Further studies on this question are needed.

We also found a significant association between changes in verbal fluency scores and right hippocampal volumes. Verbal fluency—thought to reflect semantic processing—is the ability to recall words from long-term memory in accordance with certain conditions [29]. The Jcores includes several tasks that require semantic processing and information categorization, such as combining letters to form as many words as possible and forming a word beginning with another word’s last character. Therefore, the Jcores is thought to be effective in improving verbal fluency in terms of semantic processing. The medial temporal lobe is necessary for retrieving semantically associated words, and is thus required for categorical fluency tasks. Indeed, functional neuroimaging studies in healthy individuals have reported hippocampal activation during verbal fluency tasks [30]. If CRT encourages neural activation and facilitates changes in hippocampal morphology, increased GM volume in this region might explain the observed improvements in verbal fluency.

In this study, hippocampal volume only increased on the right side. Interestingly, similar rightward lateralization patterns have also been reported in studies with healthy individuals. Kuhn et al. [16] investigated the effects of 2 months of video game training and found that the gaming group exhibited significantly greater GM increases than the control group. Similarly, Koch et al. [15] found that 14 weeks of extensive learning in medical students significantly increased right hippocampal GM volume. These results and ours suggest that hippocampus-dependent activity and/or learning affects hippocampal neuroplasticity on the right more than the left side of the brain. Future studies should investigate the laterality of activity-dependent hippocampal structure changes.

The ability of 12 weeks of CRT to increase hippocampal volume has notable implications for the neurodegenerative hypothesis of schizophrenia. Progressive brain-wide GM loss, including in the hippocampus, has been repeatedly reported in patients with schizophrenia [3]. Birchwood et al. [31] proposed that the majority of the biological, psychological, and social changes that occur in patients with schizophrenia take place in the first 5 years of disease onset; however, our participants had a mean illness duration of 12.1 years (standard deviation = 7.8 years), suggesting that clinically stable, chronically ill patients with schizophrenia also retain hippocampal plasticity. This study contributes to the development of therapeutic strategies and the understanding of the pathophysiology which underlies schizophrenia. We therefore believe that CRT for patients with schizophrenia should receive more attention as its further development will promote functional recovery.

Our study has some limitations that should be addressed. First, there exists the issue of the sample size being small. We referred to the sample size of a previous study [12], but through analysis using G*Power 3 [32], the optimal sample size would be 53 or larger (effect size f = 0.25, α error probability = 0.05 and power [1-β error probability] = 0.95). The sample size in the present study did not have sufficient power for detailed analyses, and in this respect, careful judgment is required in the interpretation of the results. We believe that additional testing with larger sample sizes is required to obtain conclusive results. Second, in this study, the intervention period was 12 weeks, and it remains necessary to verify the long-term effects of the intervention with a longer follow-up in future study. Third, ANOVA was used to compare changes in all psychometric items between groups after 12 weeks of intervention, without adjustment for the years of education and number of hospitalizations, which showed trend significance at baseline (*p* = 0.10 and 0.056, respectively). Therefore, these two psychometric items may have affected the results. Fourth, we did not control for any potential antipsychotic drug effects. The synergistic effect of neurocognitive remediation combined with antipsychotic drugs is unknown [11], and some antipsychotics may affect cerebral parenchymal volume [33]. Thus, future studies should regulate medications as much as possible. Fifth, we could not find an association between increased volumes in the right hippocampus and improved composite BACS-J scores following CRT. As this result might be due to the small sample size of this investigation, further studies with larger samples are necessary for any definitive conclusions. Sixth, we did not evaluate changes in hippocampal neural activity following CRT. We intend to use imaging technologies, including functional MRI, to address this question in the future.

## Conclusion

In summary, we found that computer-assisted CRT can promote right hippocampal GM growth in patients with schizophrenia. We also found that right hippocampal volume changes were positively correlated with alterations in verbal fluency scores. These findings enhance our understanding of hippocampal neuroplasticity and encourage the development of CRT interventions to improve cognitive and daily functions in patients with schizophrenia.

## Abbreviations

BACS-J: Brief Assessment of Cognition in Schizophrenia
BDNF: brain-derived neurotrophic factor
CRT: cognitive remediation therapy
GM: gray matter
JART: National Adult Reading Test, Japanese Version
Jcores: Japanese Cognitive Rehabilitation Program for Schizophrenia
LASMI: Life Assessment Scale for the Mentally Ill
MRI: magnetic resonance imaging
PANSS: Positive and Negative Syndrome Scale
SD: standard deviation
TAU: treatment as usual
TIV: total intracranial volume
VCAT-J: Vocational Cognitive Ability Training by Jcores
VOI: volume of interest
